# Mental health status of married women during COVID-19 pandemic in Bangladesh: A cross-sectional study

**DOI:** 10.1016/j.heliyon.2022.e08785

**Published:** 2022-01-19

**Authors:** Soumik Kha Sagar, Farhana Nusrat, Md. Utba Rashid, Prakash Ghosh, Maisha Sultana, Alvee Ahsan, Susmita Dey Pinky, Raisa Nawal Mahboob, Sajibur Rahman Nayon, Sheikh Mohammed Shariful Islam, Mohammad Delwer Hossain Hawlader

**Affiliations:** a Nutrition and Clinical Services Division (NCSD), International Centre for Diarrhoeal Disease Research, Bangladesh (icddr,b), Mohakhali, Dhaka, 1212, Bangladesh; b Sherpur Sadar Hospital, Sherpur, Mymensingh, 2100, Bangladesh; c Sylhet MAG Osmani Medical College, Medical Road, Kajolshah, Sylhet, 3100, Bangladesh; d Mymensingh Medical College, Chorpara, Mymensingh, 2200, Bangladesh; e Chattogram Medical College Hospital, Panchlaish, Chattogram, 4203, Bangladesh; f Department of Public Health, North South University, Dhaka, 1229, Bangladesh; g Public Health Professional Development Society (PPDS)

**Keywords:** Married women, Mental health, DASS-21, COVID-19, Bangladesh

## Abstract

**Aim:**

The uncontrolled spread of COVID-19 has demanded unparalleled measures, from the imposition of quarantine to the declaration as a public health emergency of international concern. COVID-19 poses a severe threat to our day-to-day life as well as physical and mental health. This study explores mental health status among married women that remain understudied in Bangladesh during the COVID-19 pandemic.

**Methodology:**

A cross-sectional study was conducted among 597 married women via face-to-face interview, maintaining all safety protocols. A semi-structured questionnaire was assembled that included socio-demographics and the DASS-21 scale. Descriptive analysis and logistic regression were performed to examine the associations between variables.

**Result:**

Almost 35% of the respondents had stress, 20% had anxiety, and 44% had depression ranging from mild to extremely severe. Metropolitan city inhabitants, being housewives, higher educational status, number of children, financial condition, comorbidities, family members assistance in household activities, relocation during COVID-19, social media use, concern about family, infected family members, tendency to get COVID-19 updates had been found significant in multivariable and univariate regression analysis with depression, anxiety, and stress.

**Conclusion:**

In this study, we found high rates of stress, anxiety, and depression among the study participants. These findings provide us with an epidemiological picture of the mental health status of our target population that could be a key benchmark for identifying high-risk groups and developing policies as well. Results could also be used to formulate psychological interventions that might be helpful during the COVID-19 period and later.

## 1 Introduction

A cluster of unexplained pneumonia cases emerged in Wuhan city, Hubei province, China, in late 2019 caused by a SARS-CoV-2 virus and spread worldwide rapidly, reaching a pandemic level. The World Health Organization (WHO) declared this as a Public Health Emergency of International Concern and designated it as Coronavirus disease 2019 (COVID-19) (Adhikari et al., 2020; Hawryluck et al., 2004; Qiu et al., 2020). SARS-CoV-2 is a highly infectious virus with tremendous transmission capacity, causing thousands of people to be infected or dead worldwide daily (World Health Organisation, 2021). The first confirmed case of COVID-19 in Bangladesh was declared in March 2020, and since then, the number of cases and deaths has been rising exponentially (DGHS, 2021). In the absence of any specific or targeted therapy, stringent public measures such as maintaining personal hygiene, social distancing, quarantine or isolation of infected or suspected individuals, locking down cities are the current mainstays to curtail the spread of this deadly virus (Adhikari et al., 2020; Medicine, 2020). Like others, the Government of Bangladesh was also compelled to enforce lockdown throughout the country initially and later shutting down selective zones where COVID-19 cases had been detected in high numbers (Tribune, 2020).

Since it was a new virus and the mode or rate of transmission was unknown, a flurry of speculation triggered confusion among the mass population, where the absence of a definitive treatment further dampened the situation with menace. This outbreak has a catastrophic impact on the economy, and the terror of this pandemic has had a detrimental effect on mental health like other infectious disease outbreaks (Hall et al., 2008; Ho et al., 2020). Moreover, the feeling of being trapped at home described as 'Pandemic-induced Claustrophobia' has resulted in erratic behavior (ex. wildly unpredictable behavior, sudden change of mood, not keeping the standard of behavior, etc.) (Ho et al., 2020). Along with quarantined or isolated people, healthy individuals also suffered from mental health issues (DiGiovanni et al., 2004; Hawryluck et al., 2004; Holmes et al., 2020; Jeong et al., 2016; Qiu et al., 2020). A recent review revealed numerous psychological and emotional outcomes, including stress, anxiety, depression, fear, confusion, frustration, boredom, etc., that persisted post-quarantine. Many studies suggested that females were more likely to suffer from psychological distress and affected by stressful events than males during this pandemic (Pfefferbaum and North, 2020).

A study performed in China reported that 35 out of 100 individuals showed psychological distress during the COVID-19 pandemic (Qiu et al., 2020). In another study in Italy, 16.5% of respondents rated moderate to severe depressive symptoms, 28.8% moderate to severe anxiety symptoms, and 8.1% reported moderate to severe stress levels (Mazza et al., 2020). Bangladesh is particularly vulnerable to this COVID-19 situation due to having one of the world's highest population densities, lack of knowledge regarding personal hygiene and reluctance among people to practice it, and poor socio-economic conditions. Compared to China and Italy, a study in Bangladesh showed a higher prevalence of stress, anxiety, and depression among the general population (59.7%, 33.7%, and 57.9%, respectively), which is supposedly due to fear of getting sick, financial constraints (fear of unemployment or significant deduction in wages) and inability to avoid venturing out for essential items (Banna et al., 2020).

On different demographics, the impact of COVID-19 on mental health is variable. Sex is considered as one of the variables that may intensify the psychological impact among the population. Previous epidemiological research placed females at a higher risk of anxiety with a profound effect on their children's health (Balhara et al., 2012; Cullen et al., 2020; Wang et al., 2020). The higher magnitude of the COVID-19 outbreak is supposed to boost their burden with lockdown effect and infection among family members. In countries like Bangladesh, married women, in general, are responsible for the overall wellbeing of their families and, in many cases, are essential wage earners. Apart from the fear of infection, concern over the health of family members and relatives, financial instability, increased amount of care work due to the dismissal of helping hand, and several other factors, including domestic abuse, contribute to the stressful condition on women. However, due to social stigma and the COVID-19 crisis, access to mental health support from family and friends or professionals has become limited, which could supposedly worsen their mental state.

Although few studies have been done to assess the mental health condition of the general population and high-risk clusters like students and healthcare workers during this COVID-19 pandemic period, no studies have yet been undertaken to assess the psychological impact of COVID-19 on females exclusively in Bangladesh. Therefore, this study aimed to assess the mental health status and associated factors among married women in Bangladesh; during this COVID-19 pandemic, which could play a key role in tailoring more effective and specific intervention and guiding policy-making to safeguard the mental health condition of this significant population.

## 2 Methodology

### 2.1 Study design and procedure

We adopted a cross-sectional survey design using a semi-structured questionnaire based on relevant literature (Banna et al., 2020; Mazza et al., 2020; Qiu et al., 2020; Sediri et al., 2020). After reviewing previous surveys, we assumed a 59% response to depression, anxiety, stress, 95% confidence level, and 5% margin of error and calculated the sample size (Banna et al., 2020). Married women (aged 18–50 years), free from mental illness, and living with family residing in Bangladesh during the COVID-19 pandemic were eligible for this study. Women who were not within the reproductive age, post-menopausal, suffering from mental illness, and critically ill were excluded from the study. Focusing on our target population, a convenience sampling strategy was utilized, and data was collected from September 15 to October 15, 2020, from the eight divisions of Bangladesh. The survey was conducted face-to-face by 26 research assistants who first explained the goals and purpose of the study to the respondents. They were asked about their menstruation history, physical and mental health conditions, whether they take any medications for their mental health problem or not. After obtaining informed consent, the data was collected by maintaining proper personal hygiene and social distancing as per governments' protocol. No financial incentive was provided to the participants, and anonymity was held to ensure the confidentiality and reliability of data.

### 2.2 Measures

We prepared a semi-structured questionnaire, which took about 10–12 min to finish. The questionnaire consists of open-ended, yes/no, multiple-choice questions and questions with a Likert scale to assess the participants' attitudes towards different aspects of this ongoing pandemic. The structured questionnaire covered several areas, including sociodemographic data, home quarantine activities, COVID-19 stressors, and the psychological impact of the COVID-19 outbreak.

Mental health measurement was done using the Depression, Anxiety, and Stress scale-21 (DASS-21) (Lovibond and Lovibond, 1995). Before this study, the DASS-21 scale was used to measure mental health state during the SARS outbreak and used in other countries as a valid and reliable way to assess mental health during COVID-19 (Ho et al., 2019; Mazza et al., 2020; Qiu et al., 2020; Quek et al., 2018; Sediri et al., 2020). This DASS-21 is a set of three self-report scales comprising 21 items, each containing seven items designed to measure the negative domain of depression, anxiety, and stress. For the convenience of our study, a validated Bangla version of DASS-21 was used, which has been proved relevant in the Bangladeshi population previously (Alim et al., 2017; Sadiq et al., 2019). We followed the Lovibond and Lovibond scoring version for categorizing depression, anxiety, and stress into our current study (Duc Tran et al., 2013).

After preparing the initial draft of the questionnaire, we validated it in two steps. Firstly, we shared the tool with the study investigators and the other researchers to give their expert opinion concerning its clarity, relativity, and significance. Secondly, a pilot study was performed by selecting a limited population (n = 40) who shared their views on simplifying and shortening the questionnaire (Cronbach's alpha values: 0.93, 0.90, and 0.93 for depression, anxiety, and stress, respectively, with an overall score of 0.96). Participants' amendments were considered and incorporated into the survey, thus maintaining continuity with contemporary literature. After a thorough discussion, the authors finalized the questionnaire (supplementary 1) and dispensed it for the study purpose.

### 2.3 Statistical analysis

Statistical analysis was performed using SPSS V25. Demographic characteristics were described as median (interquartile range, IQR) for the continuous variables and frequency for the categorical variables. We conducted a Chi-square test to analyze the association between different responses and mental health outcomes. We performed binary logistic regression to calculate the odds ratios (ORs) and the corresponding 95% confidence intervals (95% CIs) to analyze the univariate and multivariable associations between sociodemographic characteristics, home quarantine activities, COVID-19 stressors, and mental health outcomes. To perform the analysis, we estimated our cut-off value for the depression; anxiety and stress subscale were ≥10, ≥7, and ≥11, respectively, as evidence of depression, anxiety, and stress. All tests were two-sided, and p < 0.05 was regarded as statistically significant.

### 2.4 Ethical considerations

All the procedures were conducted following the ethical guidelines of the Institutional Review Board (IRB)/Ethical Review Committee (ERC) of North South University, Bangladesh (2020/OR-NSU/IRB-No.0804). The ethical standards laid down in the 1964 Declaration of Helsinki and its later amendments or comparable ethical standards were followed wherever applicable.

## 3 Result

A total of 597 participants were enrolled in this survey. Descriptive statistics of the sample are presented in Table 1. In general, respondents were from metropolitan cities (42%), aged between 26 to 35 years (55%), with a median of 29 years. More than half of the participants (60%) completed graduation or post-graduation and were from nuclear families (59%). Most of the interviewee was a housewife (48%) and having children (68%). About 52% of the respondents' families were financially solvent where domestic help for household chores was available in only 42% of the families. Only 15% of participants relocated during the COVID-19 pandemic, whereas a similar percentage did not have access to any social platforms. 42% of participants had one or more comorbidities where cardiovascular (31%) and respiratory (26%) diseases being the most prevalent ones. The majority of the participants (83%) tended to COVID-19 updates, and 71% relied on the information provided by the government.

**Table 1:** Sociodemographic characteristics of the study participants.

Characteristics	n (%)
**Age (years), median (IQR)**	29 (26–35)
**Age group**	
18–25 years	143 (24)
26–35 years	327 (55)
36–50 years	127 (21)
**Type of residential area**	
Metropolitan	253 (42)
Peri-urban	92 (15)
Suburban	48 (08)
Village	204 (34)
**Employment status**	
Student	68 (11)
Service holder	225 (38)
Housewife	286 (48)
Business	10 (02)
Other (Day laborer)	8 (01)
**Educational level**	
Never attended any school	33 (06)
Primary	96 (16)
Secondary	53 (09)
Higher secondary	60 (10)
Graduation	242 (41)
Post-graduation	113 (19)
**Parental status**	
No children	193 (32)
At least one child	148 (25)
Two children	151 (25)
More than two children	105 (18)
**Household type**	
Nuclear family	355 (59)
Joint family	76 (13)
Extended family	166 (28)
**Economic condition**	
Extremely poor	04 (1)
Poor	70 (12)
Fairly solvent	313 (52)
Quite solvent	199 (33)
Very much solvent	11 (02)
**Domestic help for household chores (Yes)**	251 (42)
**Concern over family wellbeing**	
Not concerned	31 (05)
Slightly concerned	159 (27)
Moderately concerned	147 (25)
Quite concerned	202 (34)
Extremely concerned	58 (10)
**History of COVID-19 infection (Yes)**	44 (07)
**Relocation during COVID-19 (Yes)**	89 (15)
**Job-status during lockdown**	
Same as before	412 (69)
Work from home	109 (18)
Have to give more time	32 (05)
Lost the job	44 (07)
**Use of social media**	
Do not use	87 (14)
Less	90 (15)
Same	129 (22)
Bit more	135 (23)
Much more	156 (27)
**Infected family members (Yes)**	205 (34)
**Deaths of infected family members (Yes)**	58 (10)
**Presence of chronic disease (Yes)**	251 (42)
**Types of chronic diseases**	
Allergy	06 (02)
Cardiovascular disease	77 (31)
Chronic gastritis	01 (0.4)
Chronic kidney disease	04 (02)
Chronic pancreatitis	01 (0.4)
Diabetes mellitus	35 (14)
Respiratory disease	66 (26)
Hormonal disease	14 (06)
Musculoskeletal disease	42 (17)
Obesity	02 (0.8)
Sinusitis	03 (01)
**Reliability of COVID-19 information**	
Much reliable	16 (03)
Quite reliable	120 (20)
Somewhat reliable	287 (48)
Not reliable	142 (24)
Not reliable at all	32 (05)
**Tendency to get COVID-19 updates**	
Not at all	99 (17)
Yes, daily	216 (36)
Yes, sometimes	274 (46)
Yes, many times a day	08 (01)

**Figure 1:**
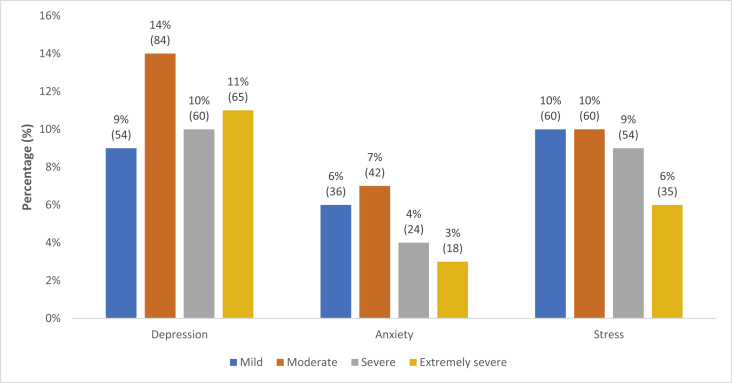
Prevalence of depression, anxiety and stress-related to COVID-19 among the married women in Bangladesh.

Among the participants, about 35% suffered from stress symptoms; however, mild (10%) and moderate (10%) symptoms were more common. Only 20% of the participants reported anxiety symptoms; among them, 7% had moderate anxiety, and 6% had mild anxiety. Almost half (44%) of the respondents experienced depressive symptoms, including moderate (14%), severe (10%), and extremely severe (12%) (Figure 1).

Factors that have association with depressive symptoms have been reported in Table 2, Table 3 and Table 4. Among the factors, higher levels of depression were associated with people living in metropolitan cities (vs village; OR = 2.3; CI: 1.6–3.4), graduate and post-graduate (vs no education; 2.0; 1.2–3.5), joint family (vs nuclear family; 1.83; 1.11–3.02), solvent (vs poor; 1.83; 1.11–3.02), co-morbid (vs no; 2.1; 1.51–2.92), relocated during COVID-19 pandemic (vs no; 1.98; 1.25–3.12), using social media (vs no; 4.5; 2.53–8.14), concerned about family (vs no; 4.4; 1.65–11.51), lost their job (vs same as before; 2.81; 1.46–5.40), family member infected (vs no; 1.60; 1.14–2.25) and affected by COVID-19 (vs no; 1.92; 1.03–3.60). Participants who were housewife (vs student; 0.49; 0.29–0.83) and had two or more than two children (vs no children; 0.45; 0.29–0.70, 0.61; 0.38–0.99) were less likely to develop any depression.

**Table 2:** Univariate association between sociodemographic variables and mental health outcomes.

Variables	Depression		Anxiety		Stress	
	P value	OR (95% CI)	P value	OR (95% CI)	P value	OR (95% CI)
**Age group**						
18–25 years		1		1		1
26–35 years	0.84	1.04 (0.70–1.55)	0.11	0.69 (0.44–1.1)	0.85	0.96 (0.64–1.45)
36–50 years	0.12	0.68 (0.42–1.11)	0.03∗	0.50 (0.27–0.91)	0.26	0.75 (0.45–1.25)
**Residential area**						
Village		1		1		1
Metropolitan city	<0.001∗∗∗	2.3 (1.6–3.4)	<0.001∗∗∗	3.4 (2.06–5.63)	<0.001∗∗∗	3.1 (2.06–4.74)
Peri urban city	0.11	1.5 (0.91–2.5)	0.76	1.1 (0.54–2.36)	0.01∗∗	2.0 (1.16–3.45)
Suburban city	0.95	0.98 (0.50–1.91)	0.04∗	2.2 (1.01–4.95)	0.07	1.9 (0.94–3.73)
**Occupation**						
Student		1		1		1
Service holder	0.344	0.77 (0.45–1.33)	0.55	0.83 (0.45–1.52)	0.83	0.94 (0.55–1.62)
Housewife	0.008∗∗	0.49 (0.29–0.83)	0.01∗∗	0.49 (0.24–0.83)	0.004∗∗	0.45 (0.26–0.78)
Business	0.139	0.34 (0.08–1.42)	0.97	0.34 (0.08–1.42)	0.81	0.84 (0.22–3.27)
Other (Day labourer)	0.117	0.26 (0.05–1.40)	1.00	--	0.12	0.18 (0.02–1.55)
**Education**						
No education		1		1		1
Primary up to higher secondary level	0.60	0.82 (0.39–1.72)	0.60	0.82 (0.39–1.72)	0.59	1.18 (0.64–2.19)
Graduation and post-graduation level	0.001∗∗∗	2.0 (1.2–3.5)	0.01∗∗	2.1 (1.2–3.5)	<0.001∗∗∗	3.0 (1.88–4.90)
**Number of children**						
No child		1		1		1
At least one child	0.568	1.13 (0.74–1.74)	0.51	0.85 (0.52–1.39)	0.81	0.95 (0.62–1.47)
Two children	<0.001∗∗∗	0.45 (0.29–0.70)	0.003∗∗	0.43 (0.24–0.75)	<0.001∗∗∗	0.42 (0.27–0.68)
More than two children	0.045∗	0.61 (0.38–0.99)	0.02∗	0.48 (0.26–0.88)	0.04∗	0.48 (0.28–0.79)
**Family type**						
Nuclear family		1		1		1
Joint family	0.018∗	1.83 (1.11–3.02)	0.02∗	1.97 (1.12–3.45)	0.11	1.52 (0.91–2.52)
Extended family	0.149	1.31 (0.91–1.91)	0.15	1.40 (0.90–2.19)	0.48	1.15 (0.78–1.70)
**Financial Condition**						
Poor		1		1		1
Solvent	0.02∗	1.83 (1.11–3.02)	0.01∗∗	2.6 (1.22–5.76)	0.001∗∗∗	2.9 (1.55–5.39)
Rich	0.60	1.31 (0.91–1.91)	0.08	2.1 (0.92–4.63)	0.04∗	1.96 (1.03–3.77)
**Comorbidities**						
**No**		1		1		1
**Yes**	<0.001∗∗∗	2.10 (1.51–2.92)	<0.001∗∗∗	2.57 (1.72–3.85)	<0.001∗∗∗	2.2 (1.58–3.14)

∗P-value< 0.05, ∗∗P-value< 0.01, ∗∗∗P-value< 0.001.

Abbreviation: OR = Odds ratio, CI = Confidence interval.

**Table 3:** Univariate association between home quarantine activities during COVID-19 and mental health outcomes.

Variables	Depression		Anxiety		Stress	
	P value	OR (95% CI)	P value	OR (95% CI)	p value	OR (95% CI)
**Domestic help for household chores**						
**No**		1		1		1
**Yes**	0.07	1.38 (0.10–1.90)	0.03∗	1.57 (1.06–2.33)	0.007∗∗	1.59 (1.13–2.24)
**Family members assistance in household activities**						
**No**		1		1		1
**Yes**	0.52	0.89 (0.62–1.27)	0.16	0.74 (0.48–1.13)	0.35	0.84 (0.57–1.22)
**Relocation during COVID-19**						
**No**		1		1		1
**Yes**	0.004∗∗	1.98 (1.25–3.12)	0.002∗∗	2.18 (1.33–3.57)	0.002∗∗	2.09 (1.32–3.29)
**Nature of living during Social Distancing period**						
Alone		1		1		1
With the family	0.46	1.17 (0.78–1.8)	0.73	1.31 (0.28–6.07)	0.57	0.72 (0.22–2.28)
Other (hostel, mess, boarding)	0.67	1.32 (0.38–4.6)	0.28	2.73 (0.44–16.75)	0.55	1.58 (0.35–6.99)
**Use of social media**						
**No**		1		1		1
**Yes**	<0.001∗∗∗	4.5 (2.53–8.14)	<0.001∗∗∗	5.1 (2.02–12.87)	<0.001∗∗∗	4.2 (2.21–8.25)

∗P-value< 0.05, ∗∗P-value< 0.01, ∗∗∗P-value< 0.001.

Abbreviation: OR = Odds ratio, CI = Confidence interval.

**Table 4:** Univariate association between COVID-19 related stressors and mental health outcomes.

Variables	Depression		Anxiety		Stress	
	P value	OR (95% CI)	p value	OR (95% CI)	p value	OR (95% CI)
**Concern about own family**						
**No**		1		1		1
**Yes**	0.001∗∗∗	4.4 (1.65–11.51)	0.04∗	8.5 (1.15–62.97)	0.02∗∗	3.7 (1.29–10.86)
**Affected by COVID-19**						
**No**		1		1		1
**Yes**	0.04∗	1.92 (1.03–3.60)	0.01∗∗	2.30 (1.20–4.40)	0.12	1.64 (0.89–3.05)
**Job-status during lockdown**						
Same as before		1		1		1
Work from home	0.539	1.14 (0.75–1.75)	0.59	1.15 (0.70–1.93)	0.42	1.20 (0.77–1.88)
Have to give more time	0.092	1.87 (0.90–3.86)	0.26	1.60 (0.71–3.59)	0.07	1.98 (0.96–4.09)
Lost the job	0.002∗∗	2.81 (1.46–5.40)	0.24	1.53 (0.76–3.10)	<0.001∗∗∗	3.24 (1.72–6.13)
**Additional social and financial support during COVID-19**						
**No**		1		1		1
**Yes**	0.27	0.8 (0.6–1.2)	0.94	1.01 (0.66–1.57)	0.66	0.92 (0.63–1.34)
**Current quarantine status**						
No		1		1		1
Yes, family	0.46	1.17 (0.78–1.6)	0.18	1.38 (0.86–2.21)	0.16	1.35 (0.89–2.05)
Yes, alone	0.67	1.31 (0.38–4.6)	1.00	1.00 (0.21–4.79)	0.83	0.86 (0.22–3.38)
**Frequency of going outside in a week**						
Do not go out		1		1		1
1-3 times	0.71	1.1 (0.72–1.61)	0.41	1.2 (0.74–2.08)	0.32	1.2 (0.81–1.91)
4-7 times	0.23	1.8 (1.1–3.0)	0.04	1.9 (1.04–3.49)	0.04	1.8 (1.04–2.98)
More than 7 times	0.34	1.3 (0.76–2.2)	0.16	1.5 (0.83–3.05)	0.26	1.4 (0.79–2.44)
**Infected family members**						
**No**		1		1		1
**Yes**	0.007∗∗	1.60 (1.14–2.25)	<0.001∗∗∗	1.96 (1.31–2.93)	0.006∗∗	1.6 (1.15–2.33)
**Death of infected family members**						
**No**		1		1		1
**Yes**	0.265	1.41 (0.82–2.42)	0.21	1.49 (0.81–2.74)	0.77	1.09 (0.62–1.91)
**Reliability of COVID-19 information**						
**Not reliable**		1		1		1
**Reliable**	0.25	0.81 (0.57–1.2)	0.17	0.74 (0.49–1.13)	0.19	0.78 (0.54–1.13)
**Tendency to get COVID-19 updates**						
**No**		1		1		1
**Yes**	0.15	0.73 (0.48–1.13)	0.001∗∗∗	0.46 (0.29–0.74)	0.12	0.70 (0.45–1.09)

∗P-value< 0.05, ∗∗P-value< 0.01, ∗∗∗P-value< 0.001.

Abbreviation: OR = Odds ratio, CI = Confidence interval.

**Table 5:** Multivariable association between variables and mental health outcomes.

	Variables	P value	Adjusted Odd Ratio	95% Confidence Interval
Depression	**Residential area (ref. village)**			
	Metropolitan city	0.008∗∗	2.06	1.21–3.52
	**Number of children (ref. no children)**			
	Two children	0.002∗∗	0.44	0.26–0.74
	**Comorbidities (ref. no)**			
	Yes	<0.001∗∗∗	2.53	1.74–3.68
	**Family members assistance in household activities (ref. no)**			
	Yes	0.010∗∗	0.56	0.34–0.87
	**Use of social media (ref. no)**			
	Yes	<0.001∗∗∗	3.55	1.87–6.76
	**Concern about own family (ref. no)**			
	Yes	0.027∗	3.20	1.14–9.00
	**Additional social and financial support during COVID-19 (ref. no)**			
	Yes	0.003∗∗	0.52	0.34–0.80
Anxiety	**Age group (ref. 18–25 years)**			
	26–35 years	0.015∗	0.53	0.32–0.89
	36–50 years	0.002∗∗	0.33	0.17–0.66
	**Residential area (ref. village)**			
	Metropolitan city	<0.001∗∗∗	3.83	2.05–7.19
	Sub-urban city	0.040∗	2.55	1.05–6.22
	**Comorbidities (ref. no)**			
	Yes	<0.001∗∗∗	3.01	1.91–4.75
	**Family members assistance in household activities (ref. no)**			
	Yes	0.003∗∗	0.44	0.25–0.75
	**Use of social media (ref. no)**			
	Yes	0.008∗∗	3.92	1.42–10.81
	**Concern about own family (ref. no)**			
	Yes	0.04∗	7.72	1.00–60.48
	**Tendency to get COVID-19 updates (ref. no)**			
	Yes	0.001∗∗∗	0.39	0.23–0.68
Stress	**Education (ref. no education)**			
	Graduation and post-graduation level	0.010∗∗	2.51	1.25–5.03
	**Comorbidities (ref. no)**			
	Yes	<0.001∗∗∗	2.25	1.54–3.27
	**Family members assistance in household activities (ref. no)**			
	Yes	0.002∗∗	0.47	0.29–0.75
	**Use of social media (ref. no)**			
	Yes	<0.001∗∗∗	3.79	1.81–7.95
	**Job status during lockdown (ref. same as before)**			
	Lost the job	0.004∗∗	2.78	1.40–5.57
	**Tendency to get COVID-19 updates (ref. no)**			
	Yes	0.024∗	0.56	0.33–0.92

Multivariable logistic regression was performed using the stepwise forward LR method.

Adjusted for age group, residential area, occupation, education, number of children, family type, financial condition, comorbidities, domestic help for household chores, family members assistance in household activities, relocating during COVID-19, nature of living during social distancing period, use of social media, concern about own family, affected by COVID-19, job status during lockdown, additional social and financial support, infected family members, death of infected family members, tendency to get COVID-19 updates.

Among the variables (Table 2), people aged between 36 to 50 years (vs 18–25 years; 0.5; 0.27–0.91), housewife (vs student; 0.49; 0.24–0.83), had two or more children (vs no children; 0.43; 0.24–0.75, 0.48; 0.26–0.88), tendency to get COVID-19 updates (vs no; 0.46; 0.29–0.74) (Table 4) had a lower odd of developing any anxiety. Whereas, participants residing in metropolitan and suburban areas (vs village; 3.4; 2.06–5.63, 2.2; 1.01–4.95), graduate and postgraduate (vs no education; 2.1; 1.2–3.5), belong to joint family (vs nuclear family; 1.97; 1.12–3.45), solvent (vs poor; 2.6; 1.22–5.76), co morbid (vs no; 2.57; 1.72–3.85) were more prone to suffer from anxiety. Again, relocation during pandemic (vs no; 2.18; 1.33–3.57), domestic help for household chores (vs no; 1.57; 1.06–2.33), use of social media (vs no; 5.1; 2.02–12.87) (Table 3), concern about family (vs no; 8.5; 1.15–62.97), affected by COVID-19 (vs no; 2.30; 1.2–4.4), infected family members (vs no; 1.96; 1.31–2.93) (Table 4) had more contribution in developing anxiety symptoms.

Factors associated with stress symptoms have been represented in Table 2, Table 3, and Table 4. Participants having two and more children (vs no children; 0.42; 0.27–0.68, 0.48; 0.28–0.79) and housewife (vs student; 0.45; 0.26–0.78) had a lower risk of stress. On the other hand, people residing in metropolitan and peri urban areas (vs village; 3.1; 2.06–4.74, 2.0; 1.16–3.45), graduate and postgraduate (vs no education; 3.0; 1.88–4.9), solvent and rich (vs poor; 2.9; 1.55–5.39, 1.96; 1-03-3.77), comorbid (vs no; 2.2; 1.58–3.14), relocated during pandemic period (vs no; 2.09; 1.32–3.29), had domestic help for household chores (vs no; 1.59; 1.13–2.24), using social media (vs no; 4.2; 2.21–8.25), had infected family members (vs no; 1.6; 1.15–2.33), concerned about family wellbeing (vs no; 3.7; 1.29–10.86), lost their job (vs same as before; 3.24; 1.72–6.13) had a greater risk of developing stress.

Multivariable logistic regression analysis (Table 5) portrayed that, after controlling the confounders, presence of comorbidities, family member's assistance in household activities, using social media during pandemic were significantly correlated with depression, anxiety, and stress among the study participants. Furthermore, patients' areas of residence and family concern were associated with creating depression and anxiety, while the tendency to get COVID-19 information was related to lowering anxiety and stress among the study respondents. Last but not least, the presence of two children in the family, different age groups (26–35 years; 36–50 years), and higher educational attainment were individually associated with depression, anxiety, and stress, respectively, among married women.

## 4 Discussion

In this study, we have investigated the mental health condition of women in Bangladesh during the COVID-19 pandemic. This is the first nationwide data on stress, anxiety, and depression targeting married females. Nearly 35% of the respondents reported suffering from stress symptoms, which are almost half (60%) than the rate found in the nationwide survey, close to India's rates (64.3%) (Banna et al., 2020; Kazmi et al., 2020). However, this rate is relatively higher than that of China (32.1%) & much higher than the UK's rates (16.8%) (Shevlin et al., 2020; Wang et al., 2020). Findings indicate that 20% of the participants had mild to extremely severe anxiety symptoms, close to the 26% reported in the nationwide survey and 28.8% reported in China. Depressive symptoms ranging from mild to extremely severe were about 44% prevalent, whereas the general population rate is 57.9% in Bangladesh, 16.5% in China, and 11.4% in Japan (Banna et al., 2020; Wang et al., 2020).

Notably, our study was conducted almost six months after the first case of COVID-19 was detected in Bangladesh, where lockdown measures had been relaxed countrywide. This could be why fewer women interviewed suffered from stress, anxiety, or depression, as information regarding the virus and its transmission was more available than in the initial stage. These findings are quite the opposite of a study done in China, where more than 70% of the population was suffering from high levels of psychological impact when the evaluation was done during the Level I Emergency Response (Tian et al., 2020). A study was conducted in Tunisia about women's mental health during the initial phase of the COVID-19 pandemic reported extremely severe anxiety (57.3%), depression (57.3%), and stress (53.1%) symptoms (Sediri et al., 2020).

In the present study, respondents residing in metropolitan cities had significant depression and anxiety that was reflected in earlier researches (Gao et al., 2020; Özdin and Bayrak Özdin, 2020). Moreover, participants who were graduate or more educated had higher rates of stress, whereas the association was significant in all three subscales among the general population in Bangladesh (Banna et al., 2020). Other studies have not found any significant differences in mental health outcomes among participants from different educational backgrounds (Jung & Jun, 2020; Zhang and Feei Ma, 2020). However, few studies reported that a lower education level is associated with higher stress, anxiety, and depression during the pandemic (Gao et al., 2020; Mazza et al., 2020; Olagoke et al., 2020; Wang et al., 2020). The greater access to information may explain these two associations and, therefore, the degree of awareness regarding COVID-19 among the educated and urban living participants, which created more psychological symptoms than those with less education or living in village areas (Zhang and Feei Ma, 2020).

This study has also found that a history of medical illness is a significant risk factor for developing psychiatric or psychological symptoms. This finding aligns with prior studies where chronic disease or self-evaluation of poor health was associated with increased psychological distress (Ho et al., 2019; Holmes et al., 2020; Wang et al., 2020). A contingent illustration of this finding could be that persons with chronic illness experience poor health due to compromised immunity and feel more vulnerable to being infected by a new disease (Hatch et al., 2018). This study has identified that social media exposure is significantly associated with higher depression, anxiety, and stress symptoms. These findings are consistent with previous studies (Gao et al., 2020; Moghanibashi-Mansourieh, 2020). The pandemic's exposure to fake news and misinformation is quickly spread via social media platforms and negatively impacts mental health (Erku et al., 2021). Some researchers suggested that seeing community members suffering from the pandemic via social media could also have adverse psychological outcomes (Li et al., 2020). In contrast, the tendency to get COVID-19 updates is associated with a lower level of anxiety and stress. The urge to get authentic news from reliable sources and get updated regarding the COVID-19 situation might be a possible reason behind lowering anxiety and stress among our study participants.

We found that concern about other family members getting COVID-19 was significantly associated with higher DASS scores in depression and anxiety subscales. This finding is also unique in Bangladesh, as studies reported only higher stress scores (Wang et al., 2020). This phenomenon could be explained by the fact that, as almost half of our study population were housewives, their concern involves more family issues than other things, which might affect the result. On the other hand, we have found a positive correlation between family members' assistance in household activities and mental health outcomes, as well as a lower level of depression, which has also been observed among participants who got social and financial support. The same finding has also been reported in previous studies (Liu et al., 2021; Qi et al., 2020). This is a clear indication that support from family and friends is a significant protective factor against psychological symptoms. Spending time with family and getting financial help from close ones help to reduce depression and improve mental health conditions. These also help individuals to maintain stable emotions during tough times.

One important finding is that people who lost their job during this period had a higher prevalence of stress. Although this is not well documented, it is pretty understandable that losing a job puts them in an ocean of uncertainty when psychological symptoms are pretty standard. We also found that women aged 18–25 had more anxiety than those aged 26–50 years, which is the opposite of a nationwide study (Banna et al., 2020). Age 18–25 is the usual time of marriage among Bangladeshi women. After marriage, they had to accommodate in a new environment at their in-laws and modify their lifestyle considerably. That could be a reason behind this finding. Besides, the health and wellbeing of the older family members could also be a reason for such observation.

In this study, we found that respondents with higher income (rich & solvent) had reported higher rates of psychological symptoms than lower-income respondents. This finding is consistent with a nationwide survey done in Bangladesh (Banna et al., 2020) but a paradox to other study reports (Lei BCD et al., 2020; Olagoke et al., 2020). Fear of living below everyday life could be a possible reason behind this finding. More qualitative exploration is needed to understand the drivers behind this finding. Another important finding of this study is that relocation or changing households during the COVID-19 period significantly impacted depression, anxiety, and stress. This phenomenon needs to be explored further but could be explained by the fact that many people were moved from big cities to their native places to cut down their expenses at the start of the lockdown measures. There was also uncertainty regarding the future situation. As this study was started just when the lockdown had been loosened, the effects of that hard time could just have been portrayed in this study.

Having an infected family member is also associated with higher DASS scores in all three subscales. This finding follows previous studies (Mazza et al., 2020; Moghanibashi-Mansourieh, 2020). Researchers reported that having an infected acquaintance is associated with increased depression and stress, whereas an infected relative increases anxiety (Mazza et al., 2020). Our findings contradict the Chinese study, which found no association among infected relative acquaintances and relatives with mental health outcomes. However, they found higher levels of concern about their family members' health (Wang et al., 2020).

To our knowledge, it is the first-ever study where we evaluated the mental health status of married women in Bangladesh during the COVID-19 pandemic. In that sense, it provides such information considering the country's cultural perspectives, which we could use to establish proper interventions in the future. And also, this study could establish a baseline on which other studies could expand on. We collected data from all the divisions of Bangladesh, which formed a large population-based study. We pilot tested the tools before data collection, which ensured the study context and setting's sustainability. Another necessary strength of this survey is that, as data collection was done by face-to-face interviews, the generalizability of collected data was ensured.

On the other hand, this study has several limitations that we must acknowledge. First, this study relied more on self-reported answers instead of mental health professionals' clinical diagnoses. So, we could not ensure alignment. Another limitation is that we did not have baseline pre-pandemic data; thus, we are not sure of any change in psychological impact levels. Lastly, due to the high surge of COVID-19 and restrictions on movement, we could not reach a large population. Notwithstanding the above limitations, this study provides invaluable information on women's psychological responses during the COVID-19 pandemic.

## 5 Conclusion

This study provides us with information regarding the burden of COVID-19 on mental health among married women in Bangladesh. Findings suggest a higher level of psychological impact, which could be a basis for developing policy aimed at women. In countries like Bangladesh, investments in the mental health sector are scarce, so improving our healthcare delivery system and strategies to protect individuals during crisis periods is essential. This starts with education and requires collaboration among families, societies, and governments. Changes would take time to set up, but strategies to policy should be there without wasting any time. Moreover, considering findings, more studies could be done in other groups to understand the burden of mental illness to set up a policy for the target population.

## Declarations

### Author contribution statement

Soumik Kha Sagar: Conceived and designed the experiments; Performed the experiments; Analyzed and interpreted the data; Contributed reagents, materials, analysis tools or data; Wrote the paper.

Farhana Nusrat, Susmita Dey Pinky, Raisa Nawal Mahboob: Conceived and designed the experiments; Performed the experiments; Contributed reagents, materials, analysis tools or data.

Md. Utba Rashid, Mohammad Delwer Hossain Hawlader: Conceived and designed the experiments; Analyzed and interpreted the data; Contributed reagents, materials, analysis tools or data; Wrote the paper.

Prakash Ghosh: Conceived and designed the experiments; Performed the experiments; Wrote the paper.

Maisha Sultana, Alvee Ahsan: Performed the experiments; Contributed reagents, materials, analysis tools or data.

Sajibur Rahman Nayon: Performed the experiments; Contributed reagents, materials, analysis tools or data; Wrote the paper.

Sheikh Mohammed Shariful Islam: Conceived and designed the experiments; Analyzed and interpreted the data; Wrote the paper.

### Funding statement

This research did not receive any specific grant from funding agencies in the public, commercial, or not-for-profit sectors.

### Data availability statement

Data will be made available on request.

### Declaration of interests statement

The authors declare no conflict of interest.

### Additional information

No additional information is available for this paper.
